# Risk Factors for Acute Kidney Injury Requiring Renal Replacement Therapy after Orthotopic Heart Transplantation in Patients with Preserved Renal Function

**DOI:** 10.3390/jcm10184117

**Published:** 2021-09-12

**Authors:** René M‘Pembele, Sebastian Roth, Alexandra Stroda, Giovanna Lurati Buse, Stephan U. Sixt, Ralf Westenfeld, Amin Polzin, Philipp Rellecke, Igor Tudorache, Markus W. Hollmann, Hug Aubin, Payam Akhyari, Artur Lichtenberg, Ragnar Huhn, Udo Boeken

**Affiliations:** 1 Department of Anesthesiology, Medical Faculty and University Hospital Duesseldorf, Heinrich-Heine-University Duesseldorf, 40225 Duesseldorf, Germany; rene.mpembele@med.uni-duesseldorf.de (R.M.); sebastian.roth@med.uni-duesseldorf.de (S.R.); alexandra.stroda@med.uni-duesseldorf.de (A.S.); giovanna.luratibuse@med.uni-duesseldorf.de (G.L.B.); stephanurs.sixt@med.uni-duesseldorf.de (S.U.S.); ragnar.huhn@med.uni-duesseldorf.de (R.H.); 2 Department of Cardiology, Pulmonology and Vascular Medicine, Medical Faculty and University Hospital Duesseldorf, Heinrich-Heine-University Duesseldorf, 40225 Duesseldorf, Germany; ralf.westenfeld@med.uni-duesseldorf.de (R.W.); amin.polzin@med.uni-duesseldorf.de (A.P.); 3 Department of Cardiac Surgery, Medical Faculty and University Hospital Duesseldorf, Heinrich-Heine-University Duesseldorf, 40225 Duesseldorf, Germany; philipp.rellecke@med.uni-duesseldorf.de (P.R.); igor.tudorache@med.uni-duesseldorf.de (I.T.); hug.aubin@med.uni-duesseldorf.de (H.A.); payam.akhyari@med.uni-duesseldorf.de (P.A.); udo.boeken@med.uni-duesseldorf.de (U.B.); 4 Amsterdam University Medical Center (AUMC), Department of Anesthesiology, Location AMC, 1105 AZ Amsterdam, The Netherlands; m.w.hollmann@amsterdamumc.nl

**Keywords:** heart failure, cardiac surgery, prognosis, vasopressors, tacrolimus, calcineurin inhibitors

## Abstract

Acute kidney injury (AKI), requiring renal replacement therapy (RRT). is a serious complication after orthotopic heart transplantation (HTX). In patients with preexisting impaired renal function, postoperative AKI is unsurprising. However, even in patients with preserved renal function, AKI requiring RRT is frequent. Therefore, this study aimed to identify risk factors associated with postoperative AKI requiring RRT after HTX in this sub-cohort. This retrospective cohort study included patients ≥ 18 years of age with preserved renal function (defined as preoperative glomerular filtration rate ≥ 60 mL/min) who underwent HTX between 2010 and 2021. In total, 107 patients were included in the analysis (mean age 52 ± 12 years, 78.5% male, 45.8% AKI requiring RRT). Based on univariate logistic regression, use of extracorporeal membrane oxygenation, postoperative infection, levosimendan therapy, duration of norepinephrine (NE) therapy and maximum daily increase in tacrolimus plasma levels were chosen to be included into multivariate analysis. Duration of NE therapy and maximum daily increase in tacrolimus plasma levels remained as independent significant risk factors (NE: OR 1.01, 95%CI: 1.00–1.02, *p* = 0.005; increase in tacrolimus plasma level: OR 1.18, 95%CI: 1.01–1.37, *p* = 0.036). In conclusion, this study identified long NE therapy and maximum daily increase in tacrolimus plasma levels as risk factors for AKI requiring RRT in HTX patients with preserved renal function.

## 1. Introduction

Acute kidney injury (AKI) is a common complication after orthotopic heart transplantation (HTX) [1,2]. A recent meta-analysis showed that incidences of AKI (according to KDIGO criteria) and AKI requiring renal replacement therapy (RRT) after HTX were 62.8% and 11.8%, respectively [3]. AKI post-HTX was associated with reduced long-term and 1-year patient survival [3,4]. In addition, AKI requiring RRT led to massive impairments regarding the patient’s quality of life [4,5]. Even in patients with preserved renal function, the occurrence of postoperative AKI requiring RRT was common [6]. Although some risk factors for postoperative AKI were previously identified, predictors for AKI in patients with preserved renal function undergoing HTX are underexplored [3]. Therefore, the aim of this study was to identify predictors for AKI requiring early RRT after HTX in patients with preserved renal function.

## 2. Materials and Methods

The present study was a retrospective, single-center cohort study and was conducted in compliance with the declaration of Helsinki and the International Society for Heart and Lung Transplantation (ISHLT) ethics statement. Ethical approval was obtained from the University of Duesseldorf’s ethic committee (Reference-number: 4567). All patients were registered in the local dedicated prospective heart transplantation database and gave written informed consent to be registered. This report follows the “Strengthening the Reporting of Observational Studies in Epidemiology” (STROBE) guidelines for cohort studies.

### 2.1. Participants

All patients ≥ 18 years of age who underwent HTX at the University Hospital Duesseldorf, Germany, between 2010 and 2021 were screened for this study. The main inclusion criterium was preserved renal function before surgery. This was defined as a glomerular filtration rate (GFR) of ≥60 mL/min calculated from creatinine clearance on the day of HTX using the “Chronic Kidney Disease Epidemiology Collaboration” (CKD-EPI) formula, according to our local laboratory standards [7]. Patients with creatinine GFR ≥ 60 mL/min but preoperative AKI requiring RRT or preoperative CKD requiring hemodialysis were excluded. Patients with missing preoperative GFR values and incomplete medical records regarding the primary endpoint were also excluded.

### 2.2. Outcome Assessment and Data Collection

The primary endpoint of this study was AKI requiring RRT within 72 h after HTX. AKI was defined according to the Kidney Disease Improving Global Outcome (KDIGO) criteria. RRT was performed as continuous veno-venous hemodialysis (CVVHD). For data collection, the local prospective HTX database was screened. Data from this database or the patient’s medical records were extracted by members of the study team. All data were double-checked by two persons trained in the study protocol.

### 2.3. Intraoperative and Postoperative Management

In our center, HTX patients are treated according to standard operating procedures. Additionally, to ensure a high quality of care, all HTX patients are treated by a small specialized team. In terms of AKI and RRT, volume management and infusion regimes may have a strong impact. Infusion regimes did not change during the study period. All patients received crystalloids as first line infusion therapy. Colloids such as hydroxyethyl starch or gelatin were not administered after heart transplantation. Albumin was only administered if the albumin plasma level was low, with a target area of 2.5–4.5 g/dL. Fresh frozen plasma, platelets and erythrocytes were given according to the cross-sectional guidelines of therapy with blood components and plasma derivatives by the German Medical Association.

### 2.4. Choice of Candidate Variables

To identify candidate variables for analysis, all variables relating to the patient, diagnosis, or associated organ dysfunction available in our database were considered. As an additional variable explored beyond the standard database contents, a maximum daily increase in tacrolimus plasma levels was separately calculated from daily tacrolimus levels. Firstly, all of these variables were assessed in a univariate analysis. As we observed 49 events in this study, a maximum of 5 covariables could be included into multivariate analysis [8]. Therefore, only significant variables in univariate analysis with good evidence of association with AKI after cardiac surgery were included into this model.

### 2.5. Statistical Analysis

All statistical analyses were performed using IBM SPSS version 25. Continuous variables are presented as means with standard deviation or median with interquartile range as appropriate. Categorical variables are presented as counts and percentages. Fisher’s exact test and *t*-tests were used to compare categorical or continuous variables for descriptive statistics. Binary logistic regression was used for univariate analysis screening of continuous or dichotomous variables, respectively. For multivariate analysis, binary multivariate logistic regression was performed to assess independent associations between chosen variables and AKI requiring RRT. A *p*-value of <0.05 was considered as significant.

## 3. Results

### 3.1. Study Cohort

**Figure 1:**
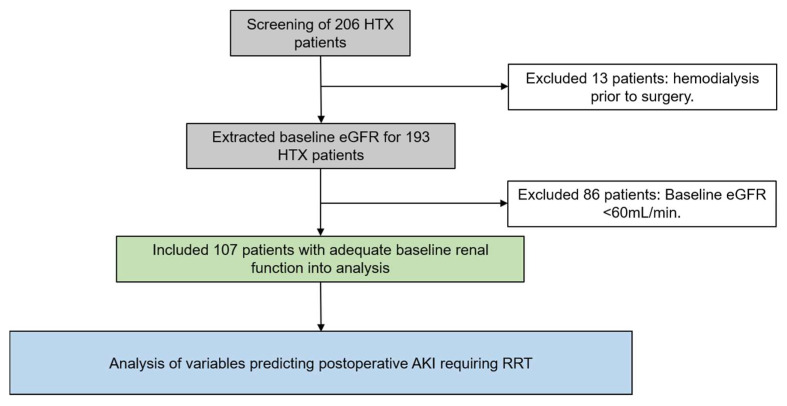
Study flow chart.

A total of 206 patients were screened for this study. A sum of 13 patients had haemodialysis prior to HTX and 86 patients had baseline GFR < 60mL/min. Based on the inclusion and exclusion criteria, 107 patients were used in the statistical analysis. Figure 1 displays selection process. The mean age of the study cohort was 52 ± 12 years and 84 patients (78.5%) were male. A total of 49 patients (45.8%) received RRT due to AKI after HTX. Detailed patient characteristics are presented in Table 1.

### 3.2. Univariate Analysis

From our local HTX database, we could assess 41 variables in univariate analysis (see Table A1). The following 9 variables were significantly associated with AKI requiring RRT in this first part of analysis (see Table 2): Post-HTX use of extracorporeal membrane oxygenation (ECMO) (OR 5.12, 95%CI: 2.07–12.67, *p* = 0.0004), post-HTX new onset of any infection (OR 3.60, 95%CI: 1.27–10.22, *p* = 0.016), post-HTX levosimendan therapy (OR 6.27, 95%CI: 2.09–18.79, *p* = 0.001), post-HTX duration of norepinephrine (NE) therapy (OR 1.01, 95%CI: 1.00–1.02, *p* = 0.002), post-HTX amount of blood products on ICU (OR 1.00, 95%CI: 1.00–1.00, *p* = 0.0004), post-HTX length of ICU stay (OR 1.04, 95%CI: 1.01–1.08, *p* = 0.008), post-HTX length of mechanical ventilation (OR 1.02, 95%CI: 1.01–1.02, *p* = < 0.0001), peak tacrolimus plasma level within first 72 h (OR 1.15, 95%CI: 1.03–1.27, *p* = 0.011), and maximum daily increase in tacrolimus plasma levels within first 72 h post-HTX (OR 1.14, 95%CI: 1.01–1.29, *p* = 0.041).

### 3.3. Multivariate Analysis

Based on the literature research, the following five of the nine significant variables were included into multivariate binary logistic regression (see Table 3): ECMO [9,10,11], all cause infection after surgery [12,13], levosimendan [14,15,16], duration of NE therapy [17,18,19] and increase in Tacrolimus plasma levels [18,20,21]. Evidence for the choice to include these five variables can be found as references next to each variable. Multivariate analysis revealed an independent significant influence of duration of NE therapy and maximum daily increase in tacrolimus plasma levels on AKI requiring RRT (NE: OR 1.01, 95%CI: 1.00–1.02, *p* = 0.005; increase in tacrolimus plasma level: OR 1.18, 95%CI: 1.01–1.37, *p* = 0.036). In addition, there was a nonsignificant trend for VA-ECMO due to primary graft dysfunction after HTX [OR 4.54, 95%CI: 0.96–21.43; *p* = 0.056].

## 4. Discussion

With our results we could show that prolonged NE therapy and maximum daily increase in tacrolimus plasma levels seem to be associated with early postoperative AKI requiring RRT after HTX in patients with preserved renal function.

### 4.1. Risk Factors for AKI Requiring RRT after HTX in the Literature

According to a recent meta-analysis, incidence of AKI is high, with up to 62.8% after HTX. Therefore, its prevention is a topic of interest for clinicians as AKI is associated with higher mortality rates [3]. Hence, the identification of risk factors for AKI after HTX was the focus of previous research. However, predictors for patients with preserved renal function is lacking. Previous cohort studies investigating HTX patients identified several patient related peri- and post-operative risk factors for AKI, as reported in the meta-analysis of Thongprayoon et al. [3]. Most of these variables were available in our database and therefore were included into univariate analysis (Table A1). Besides these variables, Euro-score, levels of Troponin I, use of Cyclosporine, right ventricular failure with higher right atrial pressure and high pulmonary vascular resistance or cardiac tamponade were reported to have association with postoperative AKI after HTX [3]. However, these variables were not accessible in our database. Only a few variables could be identified as associated with early onset AKI requiring RRT in patients with preserved renal function undergoing HTX. In line with the current literature, we can confirm that postoperative VA-ECMO therapy [22], high tacrolimus levels [20], the amount of transfusions [23], therapy with levosimendan for right ventricular failure [24], and duration of mechanical ventilation [25] were associated with AKI requiring RRT. However, out of all variables only the duration of NE therapy and maximum daily increase in tacrolimus plasma levels remained significant in our multivariable logistic regression.

### 4.2. The Role of Tacrolimus in Early AKI Requiring RRT

Tacrolimus is a crucial component of immunosuppressive therapy after HTX and is commonly started directly after surgery. However, nephrotoxicity by reduced renal blood flow is an adverse side effect, which can potentially aggravate the risk for early onset AKI [26,27]. Previous studies have already shown that high peak concentrations, above the therapeutic window of 8–12 ng/mL, are associated with AKI in post-transplant patients [18,20,21]. Sikma and co-authors demonstrated, in a retrospective cohort study including 110 patients, that supratherapeutic tacrolimus concentrations are independently associated with the development of AKI in adult HTX patients [20]. Miano and co-authors investigated early tacrolimus concentrations in 484 lung transplant recipients and also found that early tacrolimus exposure was an independent risk factor for AKI [21]. However, utility of this marker in predicting AKI is unclear as tacrolimus concentrations can be influenced by metabolic disorders which are also associated with AKI. Postoperative organ failure due to infection or sepsis could lead to impaired metabolization of tacrolimus, resulting in high plasma levels. In this context, a previous study of Percy et al. could show elevated Tacrolimus plasma levels in patients transplanted with a kidney and concomitant infection [28]. In our study, we demonstrated that tacrolimus peak plasma concentration within the first 72 h after HTX was associated with early onset AKI. Additionally, we showed that high maximum daily increase in tacrolimus plasma levels within the first 72 h was an independent predictor of AKI after HTX. In this study, postoperative infection was also associated with AKI but max. daily increase in tacrolimus plasma levels showed an independent association with AKI in multivariable logistic regression. This aspect, in addition to avoiding peak plasma concentration outside the therapeutic window, might have a significant impact in the clinical prevention of AKI after HTX. Our findings complement the limited literature in this field and should be investigated in larger, prospective trials. To avoid early postoperative AKI, alternative concepts for postoperative immunosuppressive therapy were previously proposed. In this context, calcineurin inhibitor-free induction therapy with basiliximab or anti-thymocyte globulin (ATG) showed reduced incidence of postoperative AKI after HTX as compared to calcineurin inhibitors [29]. Another concept to avoid high Tacrolimus peak plasma concentrations is use of extended-release tacrolimus. The extended release of the substance decreases the maximum concentrations while immunosuppressive effects seem to be non-inferior to regular Tacrolimus [30]. However, impact on early acute kidney injury after HTX is underexplored and should be investigated in future trials.

### 4.3. The Role of Norepinephrine in Early AKI Requiring RRT

In the present study, we found that duration of NE therapy was associated with AKI requiring RRT in patients with preserved renal function after HTX. Nephrotoxic properties of vasoactive agents by constriction of afferent renal blood vessels are critically discussed. In this context, Jocher et al. showed that elevated vasoactive inotropic score at 24 h after surgery was an independent risk factor for early onset AKI in 228 HTX patients [17]. The vasoactive inotropic score is used to objectively quantify the cardiovascular support of different vasoactive drugs. A high vasoactive inotropic score is associated with poor outcomes [31]. Interestingly, mean arterial blood pressure did not differ between AKI and non-AKI patients in the study of Jocher et al. This could lead to the conclusion that use of vasoactive drugs such as NE may directly impair renal blood flow leading to AKI. Nevertheless, adequate mean arterial blood pressure, resulting from vasoactive support, only insufficiently reflects cardiac output and organ perfusion. Microvascular perfusion can be affected by excessive vasopressors therapy to achieve mean arterial pressure goals while cardiac output remains low [32]. This aligns with our findings, where we showed an association between duration of NE therapy and AKI. Unfortunately, we were not able to assess vasoactive inotropic score. However, a major limit of vasoactive inotropic score is that it can only depict vasoactive support at one specific time point. Hence, the incidence of AKI could depend on dosing and duration of NE infusion and needs further investigation. Another study could reveal that use of dopamine, another vasoactive drug, was associated with AKI after liver transplantation [18]. However, these results are contradicting as Carrier et al. could not show any association between use of vasopressors and AKI requiring RRT after liver transplantation [33]. Once again, the postoperative use of vasoactive drugs such as NE can modify fluid balance or arise from low intravascular volume after HTX, resulting in decreased renal blood flow and therefore marks another risk factor for AKI [33,34].

### 4.4. Limitations

This study has several limitations that need to be addressed. Firstly, the incidence of early onset AKI requiring RRT in this study was high. This must be taken into account when interpreting our results. Secondly, as our sample size was rather small, we were not able to include all significant variables of the univariate model into multivariate analysis. However, some of these variables, such as prolonged ICU stay, might rather be associated with AKI than being a predictor for AKI. Thus, a larger sample size might enable the identification of more independent risk factors for AKI requiring RRT. Thirdly, this study had a retrospective design, therefore data assessment was limited to our database. Unfortunately, we could not assess vasoactive inotropic score. However, a huge amount of our data could be extracted from this prospectively conducted database, which should ensure a higher quality of data. Nevertheless, further studies should re-investigate our findings with a prospective design.

## 5. Conclusions

This study identified prolonged vasopressor therapy and high maximum daily increase in tacrolimus plasma concentrations as independent risk factors for early onset AKI requiring RRT after HTX in patients with preserved renal function. These results are clinically relevant and new therapeutic approaches for HTX patients are urgently needed. In this context, the role of calcineurin inhibitor free induction therapy or extended-release tacrolimus should be investigated.

## Figures and Tables

**Table 1:** Patient characteristics.

	All HTX Patients with Preserved Renal Function (N = 107)	HTX Patients without AKI Requiring RRT (N = 58)	HTX Patients with AKI Requiring RRT (N = 49)	*p*-Value
**Baseline characteristics**				
Male sex no. (%)	84 (78.5)	46 (79.3)	38 (77.6)	0.999
Age (years)	56 ± 12	51 ± 13	53 ± 11	0.356
Body mass index (kg/m^2^)	25.2 ± 4.9	24 ± 5	26 ± 5	0.100
**Comorbidities no. (%)**				
Arterial hypertension	62 (57.9)	34 (58.6)	28 (57.1)	0.999
Diabetes	19 (17.8)	9 (15.5)	10 (20.4)	0.614
Pulmonary hypertension	11 (10.3)	5 (8.6)	6 (12.2)	0.751
COPD	10 (10.3)	7 (12.1)	3 (6.1)	0.338
**During surgery**				
Cold ischemia time (min)	153 ± 46	156 ± 38	152 ± 54	0.635
Warm ischemia time (min)	64 ± 15	63 ± 15	65 ± 14	0.551
Overall ischemia time (min)	218 ± 46	219 ± 37	216 ± 54	0.829
Cumulative Blood product transfusion (L)	6.2 ± 4.1	5.8 ± 4.3	6.4 ± 3.9	0.492
PRBC transfusion (L)	3.8 ± 2.9	3.6 ± 2.8	3.8 ± 2.3	0.675
FFP transfusion (L)	1.5 ± 1.8	1.2 ± 1.5	1.5 ± 1.5	0.278
Thrombocyte transfusion (L)	1.1 ± 0.9	1.0 ± 0.8	1.1 ± 0.7	0.848
Duration of surgery (min)	444 ± 115	431 ± 114	459 ± 116	0.224
Duration of CPB (min)	265 ± 78	253 ± 70	279 ± 85	0.083
Duration of Reperfusion (min)	132 ± 51	126 ± 49	140 ± 53	0.165
**After surgery**				
VA-ECMO no. (%)	33 (30.8)	9 (15.8)	24 (49.0)	<0.0001
CVVHD no. (%)	49 (45.8)	0 (0)	49 (100)	<0.0001
All-cause Infection no. (%)	21 (19.6)	6 (10.9)	15 (30.6)	0.015
Resternotomy no. (%)	29 (27.1)	13 (23.6)	16 (33.3)	0.380
Days on ICU	14 (8–27)	10 (6–17)	26 (13–36)	0.002
Length of mechanical ventilation (h)	63 (25–166)	32 (18–76)	155 (49–310)	<0.0001
Cumulative Blood product transfusion (L)	9.5 ± 12.7	5.1 ± 5.4	14.6 ± 16.3	<0.0001
PRBC transfusion (L)	3.5 ± 4.3	2.1 ± 3.3	4.9 ± 5.4	0.002
FFP transfusion (L)	5.7 ± 7.1	2.6 ± 2.1	8.1 ± 9.8	<0.0001
Thrombocyte transfusion (L)	1.0 ± 2.0	0.4 ± 0.7	1.4 ± 1.9	0.001
**Medication at ICU**				
Levosimendan no. (%)	23 (21.5)	5 (9.6)	18 (40.0)	0.001
Length of epinephrine therapy (h)	134 ± 126	99 ± 87	163 ± 145	0.043
Length of norepinephrine therapy (h)	134 ± 163	73 ± 58	205 ± 57	<0.0001
Peak tacrolimus plasma level (ng/mL)	12.9 ± 5.8	12.3 ± 5.1	13.6 ± 6.6	0.281
Steepest Increase in tacrolimus plasma level (ng/mL)	6.4 ± 5.1	4.4 ± 2.4	6.8 ± 6.6	0.037
**Laboratory parameters at baseline**				
GFR (mL/min)	82.3 ± 21.8	85.8 ± 20.6	78.1 ± 22.6	0.070
Bilirubin (mg/dL)	0.7 ± 0.6	0.59 ± 0.47	0.83 ± 0.64	0.072
Albumin (g/L)	4.0 ± 0.8	3.9 ± 0.7	4.0 ± 0.8	0.787
LDH (mg/dL)	308.8 ± 222.2	281 ± 182	339 ± 258	0.210
Quick (%)	49.6 ± 26.7	51 ± 30	47 ± 22	0.456
aPTT (s)	37.9 ± 10.6	37 ± 9	39 ± 12	0.354
Hemoglobin (g/dL)	11.9 ± 2.2	12.1 ± 2.0	11.7 ± 2.3	0.309
Hematocrite (%)	36.7 ± 5.9	37.1 ± 5.5	36.2 ± 6.5	0.450

HTX = Heart Transplantation; COPD = Chronic Obstructive Pulmonary Disease; PRBC = packed red blood cells; FFP = Fresh frozen plasma; CPB = Cardiopulmonary Bypass; VA-ECMO = Veno-Arterial Extracorporeal Membrane Oxygenation; CVVHD = Continuous Veno-Venous Hemodialysis; ICU = Intensive Care Unit; GFR = Glomerular Filtration Rate; LDH = Lactate Dehydrogenase; aPTT = Activated Partial Thromboplastin Time.

**Table 2:** Univariate logistic regression for significant variables associated with acute kidney injury requiring renal replacement therapy after heart transplantation.

Variables for Univariate Logistic Regression	OR	95%CI	*p*-Value
ECMO after surgery	5.12	2.07–12.67	0.0004
All cause infection after surgery	3.60	1.27–10.22	0.016
Days at ICU	1.04	1.01–1.08	0.008
Length of mechanical ventilation	1.02	1.01–1.02	<0.0001
Cumulative blood product transfusion at ICU	1.00	1.00–1.00	0.0004
Levosimendan therapy	6.27	2.09–18.79	0.001
Duration of norepinephrine therapy	1.01	1.00–1.02	0.002
Max. daily increase in Tacrolimus plasma levels first 72 h	1.14	1.01–1.29	0.041
Tacrolimus peak concentration first 72 h	1.15	1.03–1.27	0.011

ECMO = Extracorporeal Membrane Oxygenation; ICU = Intensive Care Unit; OR = Odds Ratio; CI = Confidence Interval.

**Table 3:** Multivariate logistic regression for variables predicting acute kidney injury requiring renal replacement therapy after heart transplantation.

Variables for Multivariate Logistic Regression	Regression Coefficient	Standard Error	OR	95%CI	*p*-Value
ECMO after surgery	1.513	0.792	4.54	0.96–21.43	0.056
All cause infection after surgery	−0.339	0.948	0.71	0.11–4.57	0.720
Duration of norepinephrine therapy	0.013	0.005	1.01	1.00–1.02	0.005
Levosimendan therapy	0.154	0.870	1.17	0.21–6.42	0.860
Max. daily increase in Tacrolimus plasma level first 72 h	0.162	0.077	1.18	1.01–1.37	0.036

ECMO = Extracorporeal Membrane Oxygenation; OR = Odds Ratio; CI = Confidence Interval.

## Data Availability

All generated data can be made available on reasonable request by the first author R.M.

## Appendix A

**Table A1:** Univariate logistic regression for identification of variables associated with acute kidney injury requiring renal replacement therapy after heart transplantation.

Variables for Univariate Logistic Regression	OR	95%CI	*p*-Value
ECMO after surgery	5.12	2.07–12.67	0.0004
All cause infection after surgery	3.60	1.27–10.22	0.016
Days at ICU	1.04	1.01–1.08	0.008
Length of mechanical ventilation	1.02	1.01–1.02	<0.0001
Cumulative blood product transfusion at ICU	1.00	1.00–1.00	0.0004
Levosimendan therapy	6.27	2.09–18.79	0.001
Duration of norepinephrine therapy	1.01	1.00–1.02	0.002
Max. daily increase in Tacrolimus plasma level first 72 h	1.14	1.01–1.29	0.041
Tacrolimus peak concentration first 72 h	1.15	1.03–1.27	0.011
Duration of epinephrine therapy	1.00	1.00–1.01	0.057
Age (Recipient)	1.02	0.98–1.05	0.353
Sex (Recipient)	1.11	0.44–2.80	0.825
BMI (Recipient)	1.07	0.99–1.16	0.103
Bilirubin (Recipient)	2.27	0.92–5.58	0.075
Hemoglobin (Recipient)	0.91	0.76–1.09	0.307
Albumin (Recipient)	1.11	0.53–2.34	0.782
Prior cardiac surgery (Recipient)	1.50	0.64–3.52	0.349
Prior ventricular assist device (Recipient)	1.22	0.56–2.66	0.624
Prior resuscitation (Recipient)	1.22	0.42–3.53	0.715
Diabetes (Recipient)	1.40	0.52–3.77	0.511
Hypertension (Recipient)	0.94	0.44–2.03	0.877
Pulmonary hypertension (Recipient)	1.48	0.42–5.18	0.540
COPD (Recipient)	0.48	0.12–1.95	0.301
Age (Donor)	1.01	0.98–1.05	0.417
Sex (Donor)	0.93	0.43–2.00	0.852
BMI (Donor)	0.94	0.86–1.02	0.140
Hypertension (Donor)	1.14	0.35–3.72	0.834
Diabetes (Donor)	0.64	0.10–3.95	0.631
Creatine kinase peak (Donor)	1.00	1.00–1.00	0.461
Hemoglobin (Donor)	1.13	0.96–1.34	0.141
LVEF (Donor)	0.98	0.93–1.03	0.403
IABP after surgery	1.54	0.33–7.25	0.584
Graft rejection after surgery	0.36	0.07–1.85	0.22
CMV after surgery	0.56	0.05–6.42	0.644
Resternotomy	1.62	0.68–3.83	0.277
Duration of CPB	1.01	0.99–1.01	0.089
Duration of Reperfusion	1.01	0.99–1.01	0.172
Duration of surgery	1.00	0.99–1.01	0.224
Cold ischemic time	0.99	0.99–1.01	0.632
Warm ischemic time	1.01	0.98–1.03	0.547
Total ischemic time	0.99	0.99–1.01	0.822

ECMO = Extracorporeal Membrane Oxygenation; ICU = Intensive Care Unit; BMI = Body Mass Index; COPD = Chronic Obstructive Pulmonary Disease; LVEF = Left Ventricular Ejection Fraction; IABP = Intra-Aortic Balloon Pump; CMV = Cytmegalovirus; CPB = Cardiopulmonary Bypass; OR = Odds Ratio; CI = Confidence Interval.
